# Supplementary material for: Overlapping cell population expression profiling and regulatory inference in C. elegans
Source: BMC Genomics. 2016 Feb 29;17:159. doi: 10.1186/s12864-016-2482-z (PMC4772325; doi:10.1186/s12864-016-2482-z)
Supplement: Additional file 13: — Web supplement. (DOC 21 kb) [file 12864_2016_2482_MOESM13_ESM.zip › sortWeb/clusters/hier.300.clusters/95.html]

Cluster 95 

## Cluster 95

### Expression

| cnd-1 rep. 1 | cnd-1 rep. 2 | cnd-1 rep. 3 | pha-4 rep. 1 | pha-4 rep. 2 | pha-4 rep. 3 | ceh-27 | ceh-36 | ceh-6 | F21D5.9 | mir-57 | mls-2 | pal-1 | pros-1 | ttx-3 | unc-130 | hlh-16 | irx-1 | ceh-6 (+) hlh-16 (+) | ceh-6 (+) hlh-16 (-) | ceh-6 (-) hlh-16 (+) | cnd-1 singlets | pha-4 singlets | 0 | 60 | 120 | 150 | 180 | 240 | 330 | 390 | 420 | 480 | 540 | 570 | 600 | 630 | 660 | NAME | Functional description |
| --- | --- | --- | --- | --- | --- | --- | --- | --- | --- | --- | --- | --- | --- | --- | --- | --- | --- | --- | --- | --- | --- | --- | --- | --- | --- | --- | --- | --- | --- | --- | --- | --- | --- | --- | --- | --- | --- | --- | --- |
|  |  |  |  |  |  |  |  |  |  |  |  |  |  |  |  |  |  |  |  |  |  |  |  |  |  |  |  |  |  |  |  |  |  |  |  |  |  | *fbxa-220* | F-box A protein |
|  |  |  |  |  |  |  |  |  |  |  |  |  |  |  |  |  |  |  |  |  |  |  |  |  |  |  |  |  |  |  |  |  |  |  |  |  |  | M6.4 |  |
|  |  |  |  |  |  |  |  |  |  |  |  |  |  |  |  |  |  |  |  |  |  |  |  |  |  |  |  |  |  |  |  |  |  |  |  |  |  | *gly-18* | GLYcosylation related |
|  |  |  |  |  |  |  |  |  |  |  |  |  |  |  |  |  |  |  |  |  |  |  |  |  |  |  |  |  |  |  |  |  |  |  |  |  |  | F16B12.1 |  |
|  |  |  |  |  |  |  |  |  |  |  |  |  |  |  |  |  |  |  |  |  |  |  |  |  |  |  |  |  |  |  |  |  |  |  |  |  |  | *sup-12* | SUPpressor |
|  |  |  |  |  |  |  |  |  |  |  |  |  |  |  |  |  |  |  |  |  |  |  |  |  |  |  |  |  |  |  |  |  |  |  |  |  |  | *flp-10* | FMRF-Like Peptide |
|  |  |  |  |  |  |  |  |  |  |  |  |  |  |  |  |  |  |  |  |  |  |  |  |  |  |  |  |  |  |  |  |  |  |  |  |  |  | *ccg-1* | Conserved Cysteine/Glycine domain protein |
|  |  |  |  |  |  |  |  |  |  |  |  |  |  |  |  |  |  |  |  |  |  |  |  |  |  |  |  |  |  |  |  |  |  |  |  |  |  | *egl-15* | EGg Laying defective |
|  |  |  |  |  |  |  |  |  |  |  |  |  |  |  |  |  |  |  |  |  |  |  |  |  |  |  |  |  |  |  |  |  |  |  |  |  |  | *prk-1* | Pim (mammalian oncogene) Related Kinase |
|  |  |  |  |  |  |  |  |  |  |  |  |  |  |  |  |  |  |  |  |  |  |  |  |  |  |  |  |  |  |  |  |  |  |  |  |  |  | M7.8 |  |
|  |  |  |  |  |  |  |  |  |  |  |  |  |  |  |  |  |  |  |  |  |  |  |  |  |  |  |  |  |  |  |  |  |  |  |  |  |  | *tsp-15* | TetraSPanin family |
|  |  |  |  |  |  |  |  |  |  |  |  |  |  |  |  |  |  |  |  |  |  |  |  |  |  |  |  |  |  |  |  |  |  |  |  |  |  | ZK757.2 |  |
|  |  |  |  |  |  |  |  |  |  |  |  |  |  |  |  |  |  |  |  |  |  |  |  |  |  |  |  |  |  |  |  |  |  |  |  |  |  | *cah-5* | Carbonic AnHydrase |
|  |  |  |  |  |  |  |  |  |  |  |  |  |  |  |  |  |  |  |  |  |  |  |  |  |  |  |  |  |  |  |  |  |  |  |  |  |  | Y22D7AL.11 |  |
|  |  |  |  |  |  |  |  |  |  |  |  |  |  |  |  |  |  |  |  |  |  |  |  |  |  |  |  |  |  |  |  |  |  |  |  |  |  | *ric-7* | Resistance to Inhibitors of Cholinesterase |
|  |  |  |  |  |  |  |  |  |  |  |  |  |  |  |  |  |  |  |  |  |  |  |  |  |  |  |  |  |  |  |  |  |  |  |  |  |  | T01E8.1 |  |
|  |  |  |  |  |  |  |  |  |  |  |  |  |  |  |  |  |  |  |  |  |  |  |  |  |  |  |  |  |  |  |  |  |  |  |  |  |  | *mct-3* | MonoCarboxylate Transporter family |
|  |  |  |  |  |  |  |  |  |  |  |  |  |  |  |  |  |  |  |  |  |  |  |  |  |  |  |  |  |  |  |  |  |  |  |  |  |  | Y53F4B.45 |  |
|  |  |  |  |  |  |  |  |  |  |  |  |  |  |  |  |  |  |  |  |  |  |  |  |  |  |  |  |  |  |  |  |  |  |  |  |  |  | *ttr-53* | TransThyretin-Related family domain |
|  |  |  |  |  |  |  |  |  |  |  |  |  |  |  |  |  |  |  |  |  |  |  |  |  |  |  |  |  |  |  |  |  |  |  |  |  |  | F08G5.3 |  |
|  |  |  |  |  |  |  |  |  |  |  |  |  |  |  |  |  |  |  |  |  |  |  |  |  |  |  |  |  |  |  |  |  |  |  |  |  |  | T07F12.4 |  |
|  |  |  |  |  |  |  |  |  |  |  |  |  |  |  |  |  |  |  |  |  |  |  |  |  |  |  |  |  |  |  |  |  |  |  |  |  |  | *aakg-3* | AMP-Activated protein Kinase Gamma subunit |
|  |  |  |  |  |  |  |  |  |  |  |  |  |  |  |  |  |  |  |  |  |  |  |  |  |  |  |  |  |  |  |  |  |  |  |  |  |  | *lec-2* | gaLECtin |
|  |  |  |  |  |  |  |  |  |  |  |  |  |  |  |  |  |  |  |  |  |  |  |  |  |  |  |  |  |  |  |  |  |  |  |  |  |  | *glb-1* | GLoBin related |
|  |  |  |  |  |  |  |  |  |  |  |  |  |  |  |  |  |  |  |  |  |  |  |  |  |  |  |  |  |  |  |  |  |  |  |  |  |  | F42G10.1 |  |
|  |  |  |  |  |  |  |  |  |  |  |  |  |  |  |  |  |  |  |  |  |  |  |  |  |  |  |  |  |  |  |  |  |  |  |  |  |  | C44B7.6 |  |
|  |  |  |  |  |  |  |  |  |  |  |  |  |  |  |  |  |  |  |  |  |  |  |  |  |  |  |  |  |  |  |  |  |  |  |  |  |  | *nhr-187* | Nuclear Hormone Receptor family |
|  |  |  |  |  |  |  |  |  |  |  |  |  |  |  |  |  |  |  |  |  |  |  |  |  |  |  |  |  |  |  |  |  |  |  |  |  |  | *tag-273* | Temporarily Assigned Gene name |
|  |  |  |  |  |  |  |  |  |  |  |  |  |  |  |  |  |  |  |  |  |  |  |  |  |  |  |  |  |  |  |  |  |  |  |  |  |  | *unc-93* | UNCoordinated |
|  |  |  |  |  |  |  |  |  |  |  |  |  |  |  |  |  |  |  |  |  |  |  |  |  |  |  |  |  |  |  |  |  |  |  |  |  |  | T23E7.2 |  |
|  |  |  |  |  |  |  |  |  |  |  |  |  |  |  |  |  |  |  |  |  |  |  |  |  |  |  |  |  |  |  |  |  |  |  |  |  |  | *dig-1* | DIsplaced Gonad |
|  |  |  |  |  |  |  |  |  |  |  |  |  |  |  |  |  |  |  |  |  |  |  |  |  |  |  |  |  |  |  |  |  |  |  |  |  |  | F46G10.2 |  |
|  |  |  |  |  |  |  |  |  |  |  |  |  |  |  |  |  |  |  |  |  |  |  |  |  |  |  |  |  |  |  |  |  |  |  |  |  |  | K10B3.6 |  |
|  |  |  |  |  |  |  |  |  |  |  |  |  |  |  |  |  |  |  |  |  |  |  |  |  |  |  |  |  |  |  |  |  |  |  |  |  |  | *hpo-28* | Hypersensitive to POre-forming toxin |
|  |  |  |  |  |  |  |  |  |  |  |  |  |  |  |  |  |  |  |  |  |  |  |  |  |  |  |  |  |  |  |  |  |  |  |  |  |  | R11G1.6 |  |
|  |  |  |  |  |  |  |  |  |  |  |  |  |  |  |  |  |  |  |  |  |  |  |  |  |  |  |  |  |  |  |  |  |  |  |  |  |  | C02D5.2 |  |
|  |  |  |  |  |  |  |  |  |  |  |  |  |  |  |  |  |  |  |  |  |  |  |  |  |  |  |  |  |  |  |  |  |  |  |  |  |  | C33D9.8 |  |
|  |  |  |  |  |  |  |  |  |  |  |  |  |  |  |  |  |  |  |  |  |  |  |  |  |  |  |  |  |  |  |  |  |  |  |  |  |  | *fbp-1* | Fructose-1,6-BiPhosphatase |
|  |  |  |  |  |  |  |  |  |  |  |  |  |  |  |  |  |  |  |  |  |  |  |  |  |  |  |  |  |  |  |  |  |  |  |  |  |  | C49A1.10 |  |
|  |  |  |  |  |  |  |  |  |  |  |  |  |  |  |  |  |  |  |  |  |  |  |  |  |  |  |  |  |  |  |  |  |  |  |  |  |  | *srp-1* | SeRPin |
|  |  |  |  |  |  |  |  |  |  |  |  |  |  |  |  |  |  |  |  |  |  |  |  |  |  |  |  |  |  |  |  |  |  |  |  |  |  | F22E10.7 |  |
|  |  |  |  |  |  |  |  |  |  |  |  |  |  |  |  |  |  |  |  |  |  |  |  |  |  |  |  |  |  |  |  |  |  |  |  |  |  | *ark-1* | A Ras-regulating Kinase |
|  |  |  |  |  |  |  |  |  |  |  |  |  |  |  |  |  |  |  |  |  |  |  |  |  |  |  |  |  |  |  |  |  |  |  |  |  |  | C35C5.16 |  |
|  |  |  |  |  |  |  |  |  |  |  |  |  |  |  |  |  |  |  |  |  |  |  |  |  |  |  |  |  |  |  |  |  |  |  |  |  |  | Y48B6A.5 |  |
|  |  |  |  |  |  |  |  |  |  |  |  |  |  |  |  |  |  |  |  |  |  |  |  |  |  |  |  |  |  |  |  |  |  |  |  |  |  | T06C10.3 |  |
|  |  |  |  |  |  |  |  |  |  |  |  |  |  |  |  |  |  |  |  |  |  |  |  |  |  |  |  |  |  |  |  |  |  |  |  |  |  | *sri-19* | Serpentine Receptor, class I |
|  |  |  |  |  |  |  |  |  |  |  |  |  |  |  |  |  |  |  |  |  |  |  |  |  |  |  |  |  |  |  |  |  |  |  |  |  |  | B0563.9 |  |
|  |  |  |  |  |  |  |  |  |  |  |  |  |  |  |  |  |  |  |  |  |  |  |  |  |  |  |  |  |  |  |  |  |  |  |  |  |  | *klo-2* | KLOtho (mammalian aging-associated protein) homolog |
|  |  |  |  |  |  |  |  |  |  |  |  |  |  |  |  |  |  |  |  |  |  |  |  |  |  |  |  |  |  |  |  |  |  |  |  |  |  | *ndnf-1* | NDNF (Neuron Derived Neurotrophic Factor) homolog |
|  |  |  |  |  |  |  |  |  |  |  |  |  |  |  |  |  |  |  |  |  |  |  |  |  |  |  |  |  |  |  |  |  |  |  |  |  |  | *pck-1* | Phosphoenolypyruvate CarboxyKinase |
|  |  |  |  |  |  |  |  |  |  |  |  |  |  |  |  |  |  |  |  |  |  |  |  |  |  |  |  |  |  |  |  |  |  |  |  |  |  | *fln-1* | FiLamiN (actin binding protein) homolog |
|  |  |  |  |  |  |  |  |  |  |  |  |  |  |  |  |  |  |  |  |  |  |  |  |  |  |  |  |  |  |  |  |  |  |  |  |  |  | *pqn-52* | Prion-like-(Q/N-rich)-domain-bearing protein |
|  |  |  |  |  |  |  |  |  |  |  |  |  |  |  |  |  |  |  |  |  |  |  |  |  |  |  |  |  |  |  |  |  |  |  |  |  |  | *molo-1* | MOdulator of LevamisOle-sensitive receptor |
|  |  |  |  |  |  |  |  |  |  |  |  |  |  |  |  |  |  |  |  |  |  |  |  |  |  |  |  |  |  |  |  |  |  |  |  |  |  | F53C3.8 |  |
|  |  |  |  |  |  |  |  |  |  |  |  |  |  |  |  |  |  |  |  |  |  |  |  |  |  |  |  |  |  |  |  |  |  |  |  |  |  | Y55B1BR.6 |  |
|  |  |  |  |  |  |  |  |  |  |  |  |  |  |  |  |  |  |  |  |  |  |  |  |  |  |  |  |  |  |  |  |  |  |  |  |  |  | *inx-18* | INneXin |
|  |  |  |  |  |  |  |  |  |  |  |  |  |  |  |  |  |  |  |  |  |  |  |  |  |  |  |  |  |  |  |  |  |  |  |  |  |  | *tsp-8* | TetraSPanin family |
|  |  |  |  |  |  |  |  |  |  |  |  |  |  |  |  |  |  |  |  |  |  |  |  |  |  |  |  |  |  |  |  |  |  |  |  |  |  | C02C2.5 |  |
|  |  |  |  |  |  |  |  |  |  |  |  |  |  |  |  |  |  |  |  |  |  |  |  |  |  |  |  |  |  |  |  |  |  |  |  |  |  | Y45F10B.13 |  |
|  |  |  |  |  |  |  |  |  |  |  |  |  |  |  |  |  |  |  |  |  |  |  |  |  |  |  |  |  |  |  |  |  |  |  |  |  |  | *tnt-2* | TropoNin T |
|  |  |  |  |  |  |  |  |  |  |  |  |  |  |  |  |  |  |  |  |  |  |  |  |  |  |  |  |  |  |  |  |  |  |  |  |  |  | F40F8.5 |  |
|  |  |  |  |  |  |  |  |  |  |  |  |  |  |  |  |  |  |  |  |  |  |  |  |  |  |  |  |  |  |  |  |  |  |  |  |  |  | *lev-9* | LEVamisole resistant |
|  |  |  |  |  |  |  |  |  |  |  |  |  |  |  |  |  |  |  |  |  |  |  |  |  |  |  |  |  |  |  |  |  |  |  |  |  |  | T07H6.4 |  |
|  |  |  |  |  |  |  |  |  |  |  |  |  |  |  |  |  |  |  |  |  |  |  |  |  |  |  |  |  |  |  |  |  |  |  |  |  |  | *ace-1* | abnormal AcetylCholinEsterase |
|  |  |  |  |  |  |  |  |  |  |  |  |  |  |  |  |  |  |  |  |  |  |  |  |  |  |  |  |  |  |  |  |  |  |  |  |  |  | T21B6.3 |  |
|  |  |  |  |  |  |  |  |  |  |  |  |  |  |  |  |  |  |  |  |  |  |  |  |  |  |  |  |  |  |  |  |  |  |  |  |  |  | ZC374.2 |  |
|  |  |  |  |  |  |  |  |  |  |  |  |  |  |  |  |  |  |  |  |  |  |  |  |  |  |  |  |  |  |  |  |  |  |  |  |  |  | *lgc-34* | Ligand-Gated ion Channel |
|  |  |  |  |  |  |  |  |  |  |  |  |  |  |  |  |  |  |  |  |  |  |  |  |  |  |  |  |  |  |  |  |  |  |  |  |  |  | F09B9.4 |  |
|  |  |  |  |  |  |  |  |  |  |  |  |  |  |  |  |  |  |  |  |  |  |  |  |  |  |  |  |  |  |  |  |  |  |  |  |  |  | T03G6.3 |  |
|  |  |  |  |  |  |  |  |  |  |  |  |  |  |  |  |  |  |  |  |  |  |  |  |  |  |  |  |  |  |  |  |  |  |  |  |  |  | *unc-87* | UNCoordinated |
|  |  |  |  |  |  |  |  |  |  |  |  |  |  |  |  |  |  |  |  |  |  |  |  |  |  |  |  |  |  |  |  |  |  |  |  |  |  | T07H6.1 |  |
|  |  |  |  |  |  |  |  |  |  |  |  |  |  |  |  |  |  |  |  |  |  |  |  |  |  |  |  |  |  |  |  |  |  |  |  |  |  | *csq-1* | CalSeQuestrin |
|  |  |  |  |  |  |  |  |  |  |  |  |  |  |  |  |  |  |  |  |  |  |  |  |  |  |  |  |  |  |  |  |  |  |  |  |  |  | F47B3.3 |  |
|  |  |  |  |  |  |  |  |  |  |  |  |  |  |  |  |  |  |  |  |  |  |  |  |  |  |  |  |  |  |  |  |  |  |  |  |  |  | F54D12.5 |  |
|  |  |  |  |  |  |  |  |  |  |  |  |  |  |  |  |  |  |  |  |  |  |  |  |  |  |  |  |  |  |  |  |  |  |  |  |  |  | *acs-16* | fatty Acid CoA Synthetase family |
|  |  |  |  |  |  |  |  |  |  |  |  |  |  |  |  |  |  |  |  |  |  |  |  |  |  |  |  |  |  |  |  |  |  |  |  |  |  | *ncs-4* | Neuronal Calcium Sensor family |
|  |  |  |  |  |  |  |  |  |  |  |  |  |  |  |  |  |  |  |  |  |  |  |  |  |  |  |  |  |  |  |  |  |  |  |  |  |  | C51E3.9 |  |
|  |  |  |  |  |  |  |  |  |  |  |  |  |  |  |  |  |  |  |  |  |  |  |  |  |  |  |  |  |  |  |  |  |  |  |  |  |  | *srsx-26* | Serpentine Receptor, class SX |
|  |  |  |  |  |  |  |  |  |  |  |  |  |  |  |  |  |  |  |  |  |  |  |  |  |  |  |  |  |  |  |  |  |  |  |  |  |  | T28D9.3 |  |
|  |  |  |  |  |  |  |  |  |  |  |  |  |  |  |  |  |  |  |  |  |  |  |  |  |  |  |  |  |  |  |  |  |  |  |  |  |  | *ttr-47* | TransThyretin-Related family domain |
|  |  |  |  |  |  |  |  |  |  |  |  |  |  |  |  |  |  |  |  |  |  |  |  |  |  |  |  |  |  |  |  |  |  |  |  |  |  | VC5.2 |  |
|  |  |  |  |  |  |  |  |  |  |  |  |  |  |  |  |  |  |  |  |  |  |  |  |  |  |  |  |  |  |  |  |  |  |  |  |  |  | *ttr-16* | TransThyretin-Related family domain |
|  |  |  |  |  |  |  |  |  |  |  |  |  |  |  |  |  |  |  |  |  |  |  |  |  |  |  |  |  |  |  |  |  |  |  |  |  |  | *ttr-17* | TransThyretin-Related family domain |
|  |  |  |  |  |  |  |  |  |  |  |  |  |  |  |  |  |  |  |  |  |  |  |  |  |  |  |  |  |  |  |  |  |  |  |  |  |  | *lec-5* | gaLECtin |
|  |  |  |  |  |  |  |  |  |  |  |  |  |  |  |  |  |  |  |  |  |  |  |  |  |  |  |  |  |  |  |  |  |  |  |  |  |  | *mec-5* | MEChanosensory abnormality |
|  |  |  |  |  |  |  |  |  |  |  |  |  |  |  |  |  |  |  |  |  |  |  |  |  |  |  |  |  |  |  |  |  |  |  |  |  |  | F10A3.11 |  |
|  |  |  |  |  |  |  |  |  |  |  |  |  |  |  |  |  |  |  |  |  |  |  |  |  |  |  |  |  |  |  |  |  |  |  |  |  |  | *ckr-2* | CholecystoKinin Receptor homolog |
|  |  |  |  |  |  |  |  |  |  |  |  |  |  |  |  |  |  |  |  |  |  |  |  |  |  |  |  |  |  |  |  |  |  |  |  |  |  | R04B5.5 |  |
|  |  |  |  |  |  |  |  |  |  |  |  |  |  |  |  |  |  |  |  |  |  |  |  |  |  |  |  |  |  |  |  |  |  |  |  |  |  | *cal-1* | CALmodulin related genes |
|  |  |  |  |  |  |  |  |  |  |  |  |  |  |  |  |  |  |  |  |  |  |  |  |  |  |  |  |  |  |  |  |  |  |  |  |  |  | T28B4.1 |  |
|  |  |  |  |  |  |  |  |  |  |  |  |  |  |  |  |  |  |  |  |  |  |  |  |  |  |  |  |  |  |  |  |  |  |  |  |  |  | *oig-2* | One IG domain |
|  |  |  |  |  |  |  |  |  |  |  |  |  |  |  |  |  |  |  |  |  |  |  |  |  |  |  |  |  |  |  |  |  |  |  |  |  |  | *tag-243* | Temporarily Assigned Gene name |
|  |  |  |  |  |  |  |  |  |  |  |  |  |  |  |  |  |  |  |  |  |  |  |  |  |  |  |  |  |  |  |  |  |  |  |  |  |  | T02D1.8 |  |
|  |  |  |  |  |  |  |  |  |  |  |  |  |  |  |  |  |  |  |  |  |  |  |  |  |  |  |  |  |  |  |  |  |  |  |  |  |  | F08F3.10 |  |
|  |  |  |  |  |  |  |  |  |  |  |  |  |  |  |  |  |  |  |  |  |  |  |  |  |  |  |  |  |  |  |  |  |  |  |  |  |  | K08C7.6 |  |
|  |  |  |  |  |  |  |  |  |  |  |  |  |  |  |  |  |  |  |  |  |  |  |  |  |  |  |  |  |  |  |  |  |  |  |  |  |  | F10G2.1 |  |
|  |  |  |  |  |  |  |  |  |  |  |  |  |  |  |  |  |  |  |  |  |  |  |  |  |  |  |  |  |  |  |  |  |  |  |  |  |  | M02D8.6 |  |
|  |  |  |  |  |  |  |  |  |  |  |  |  |  |  |  |  |  |  |  |  |  |  |  |  |  |  |  |  |  |  |  |  |  |  |  |  |  | T05E7.4 |  |
|  |  |  |  |  |  |  |  |  |  |  |  |  |  |  |  |  |  |  |  |  |  |  |  |  |  |  |  |  |  |  |  |  |  |  |  |  |  | R12C12.9 |  |
|  |  |  |  |  |  |  |  |  |  |  |  |  |  |  |  |  |  |  |  |  |  |  |  |  |  |  |  |  |  |  |  |  |  |  |  |  |  | *col-110* | COLlagen |
|  |  |  |  |  |  |  |  |  |  |  |  |  |  |  |  |  |  |  |  |  |  |  |  |  |  |  |  |  |  |  |  |  |  |  |  |  |  | C54F6.5 |  |
|  |  |  |  |  |  |  |  |  |  |  |  |  |  |  |  |  |  |  |  |  |  |  |  |  |  |  |  |  |  |  |  |  |  |  |  |  |  | C15A11.2 |  |
|  |  |  |  |  |  |  |  |  |  |  |  |  |  |  |  |  |  |  |  |  |  |  |  |  |  |  |  |  |  |  |  |  |  |  |  |  |  | F54D12.4 |  |
|  |  |  |  |  |  |  |  |  |  |  |  |  |  |  |  |  |  |  |  |  |  |  |  |  |  |  |  |  |  |  |  |  |  |  |  |  |  | EGAP4.1 |  |
|  |  |  |  |  |  |  |  |  |  |  |  |  |  |  |  |  |  |  |  |  |  |  |  |  |  |  |  |  |  |  |  |  |  |  |  |  |  | K06A9.3 |  |
|  |  |  |  |  |  |  |  |  |  |  |  |  |  |  |  |  |  |  |  |  |  |  |  |  |  |  |  |  |  |  |  |  |  |  |  |  |  | *srd-53* | Serpentine Receptor, class D (delta) |
|  |  |  |  |  |  |  |  |  |  |  |  |  |  |  |  |  |  |  |  |  |  |  |  |  |  |  |  |  |  |  |  |  |  |  |  |  |  | F13G3.12 |  |
|  |  |  |  |  |  |  |  |  |  |  |  |  |  |  |  |  |  |  |  |  |  |  |  |  |  |  |  |  |  |  |  |  |  |  |  |  |  | *del-6* | DEgenerin Like |
|  |  |  |  |  |  |  |  |  |  |  |  |  |  |  |  |  |  |  |  |  |  |  |  |  |  |  |  |  |  |  |  |  |  |  |  |  |  | *anr-28* | Antisense Non-coding RNA |
|  |  |  |  |  |  |  |  |  |  |  |  |  |  |  |  |  |  |  |  |  |  |  |  |  |  |  |  |  |  |  |  |  |  |  |  |  |  | *anr-26* | Antisense Non-coding RNA |
|  |  |  |  |  |  |  |  |  |  |  |  |  |  |  |  |  |  |  |  |  |  |  |  |  |  |  |  |  |  |  |  |  |  |  |  |  |  | T03E6.9 |  |
|  |  |  |  |  |  |  |  |  |  |  |  |  |  |  |  |  |  |  |  |  |  |  |  |  |  |  |  |  |  |  |  |  |  |  |  |  |  | F19C7.2 |  |
|  |  |  |  |  |  |  |  |  |  |  |  |  |  |  |  |  |  |  |  |  |  |  |  |  |  |  |  |  |  |  |  |  |  |  |  |  |  | C39B10.3 |  |
|  |  |  |  |  |  |  |  |  |  |  |  |  |  |  |  |  |  |  |  |  |  |  |  |  |  |  |  |  |  |  |  |  |  |  |  |  |  | C27B7.7 |  |
|  |  |  |  |  |  |  |  |  |  |  |  |  |  |  |  |  |  |  |  |  |  |  |  |  |  |  |  |  |  |  |  |  |  |  |  |  |  | ZK822.1 |  |
|  |  |  |  |  |  |  |  |  |  |  |  |  |  |  |  |  |  |  |  |  |  |  |  |  |  |  |  |  |  |  |  |  |  |  |  |  |  | *tag-278* | Temporarily Assigned Gene name |
|  |  |  |  |  |  |  |  |  |  |  |  |  |  |  |  |  |  |  |  |  |  |  |  |  |  |  |  |  |  |  |  |  |  |  |  |  |  | *tmc-2* | TMC (TransMembrane Channel-like protein) family |
|  |  |  |  |  |  |  |  |  |  |  |  |  |  |  |  |  |  |  |  |  |  |  |  |  |  |  |  |  |  |  |  |  |  |  |  |  |  | *gsnl-1* | GelSoliN-Like |
|  |  |  |  |  |  |  |  |  |  |  |  |  |  |  |  |  |  |  |  |  |  |  |  |  |  |  |  |  |  |  |  |  |  |  |  |  |  | T10G3.1 |  |
|  |  |  |  |  |  |  |  |  |  |  |  |  |  |  |  |  |  |  |  |  |  |  |  |  |  |  |  |  |  |  |  |  |  |  |  |  |  | *pqn-24* | Prion-like-(Q/N-rich)-domain-bearing protein |
|  |  |  |  |  |  |  |  |  |  |  |  |  |  |  |  |  |  |  |  |  |  |  |  |  |  |  |  |  |  |  |  |  |  |  |  |  |  | *zig-7* | 2 (Zwei) IG domain protein |
|  |  |  |  |  |  |  |  |  |  |  |  |  |  |  |  |  |  |  |  |  |  |  |  |  |  |  |  |  |  |  |  |  |  |  |  |  |  | F11F1.6 |  |
|  |  |  |  |  |  |  |  |  |  |  |  |  |  |  |  |  |  |  |  |  |  |  |  |  |  |  |  |  |  |  |  |  |  |  |  |  |  | Y9C2UA.1 |  |
|  |  |  |  |  |  |  |  |  |  |  |  |  |  |  |  |  |  |  |  |  |  |  |  |  |  |  |  |  |  |  |  |  |  |  |  |  |  | C46G7.2 |  |
|  |  |  |  |  |  |  |  |  |  |  |  |  |  |  |  |  |  |  |  |  |  |  |  |  |  |  |  |  |  |  |  |  |  |  |  |  |  | *acs-17* | fatty Acid CoA Synthetase family |
|  |  |  |  |  |  |  |  |  |  |  |  |  |  |  |  |  |  |  |  |  |  |  |  |  |  |  |  |  |  |  |  |  |  |  |  |  |  | Y37D8A.2 |  |
|  |  |  |  |  |  |  |  |  |  |  |  |  |  |  |  |  |  |  |  |  |  |  |  |  |  |  |  |  |  |  |  |  |  |  |  |  |  | *twk-31* | TWiK family of potassium channels |
|  |  |  |  |  |  |  |  |  |  |  |  |  |  |  |  |  |  |  |  |  |  |  |  |  |  |  |  |  |  |  |  |  |  |  |  |  |  | *twk-18* | TWiK family of potassium channels |
|  |  |  |  |  |  |  |  |  |  |  |  |  |  |  |  |  |  |  |  |  |  |  |  |  |  |  |  |  |  |  |  |  |  |  |  |  |  | *unc-29* | UNCoordinated |
|  |  |  |  |  |  |  |  |  |  |  |  |  |  |  |  |  |  |  |  |  |  |  |  |  |  |  |  |  |  |  |  |  |  |  |  |  |  | *alh-12* | ALdehyde deHydrogenase |
|  |  |  |  |  |  |  |  |  |  |  |  |  |  |  |  |  |  |  |  |  |  |  |  |  |  |  |  |  |  |  |  |  |  |  |  |  |  | *stn-2* | SynTrophiN |
|  |  |  |  |  |  |  |  |  |  |  |  |  |  |  |  |  |  |  |  |  |  |  |  |  |  |  |  |  |  |  |  |  |  |  |  |  |  | W03G11.3 |  |
|  |  |  |  |  |  |  |  |  |  |  |  |  |  |  |  |  |  |  |  |  |  |  |  |  |  |  |  |  |  |  |  |  |  |  |  |  |  | *cpna-1* | CoPiNe domain protein, Atypical |
|  |  |  |  |  |  |  |  |  |  |  |  |  |  |  |  |  |  |  |  |  |  |  |  |  |  |  |  |  |  |  |  |  |  |  |  |  |  | Y69A2AR.31 |  |
|  |  |  |  |  |  |  |  |  |  |  |  |  |  |  |  |  |  |  |  |  |  |  |  |  |  |  |  |  |  |  |  |  |  |  |  |  |  | *doxa-1* | dual oxidase maturation factor |
|  |  |  |  |  |  |  |  |  |  |  |  |  |  |  |  |  |  |  |  |  |  |  |  |  |  |  |  |  |  |  |  |  |  |  |  |  |  | DH11.2 |  |
|  |  |  |  |  |  |  |  |  |  |  |  |  |  |  |  |  |  |  |  |  |  |  |  |  |  |  |  |  |  |  |  |  |  |  |  |  |  | R31.2 |  |
|  |  |  |  |  |  |  |  |  |  |  |  |  |  |  |  |  |  |  |  |  |  |  |  |  |  |  |  |  |  |  |  |  |  |  |  |  |  | Y55D5A.3 |  |
|  |  |  |  |  |  |  |  |  |  |  |  |  |  |  |  |  |  |  |  |  |  |  |  |  |  |  |  |  |  |  |  |  |  |  |  |  |  | *mig-17* | abnormal cell MIGration |
|  |  |  |  |  |  |  |  |  |  |  |  |  |  |  |  |  |  |  |  |  |  |  |  |  |  |  |  |  |  |  |  |  |  |  |  |  |  | *twk-28* | TWiK family of potassium channels |
|  |  |  |  |  |  |  |  |  |  |  |  |  |  |  |  |  |  |  |  |  |  |  |  |  |  |  |  |  |  |  |  |  |  |  |  |  |  | *gst-22* | Glutathione S-Transferase |
|  |  |  |  |  |  |  |  |  |  |  |  |  |  |  |  |  |  |  |  |  |  |  |  |  |  |  |  |  |  |  |  |  |  |  |  |  |  | R151.4 |  |
|  |  |  |  |  |  |  |  |  |  |  |  |  |  |  |  |  |  |  |  |  |  |  |  |  |  |  |  |  |  |  |  |  |  |  |  |  |  | W06A7.2 |  |
|  |  |  |  |  |  |  |  |  |  |  |  |  |  |  |  |  |  |  |  |  |  |  |  |  |  |  |  |  |  |  |  |  |  |  |  |  |  | Y55B1BR.5 |  |
|  |  |  |  |  |  |  |  |  |  |  |  |  |  |  |  |  |  |  |  |  |  |  |  |  |  |  |  |  |  |  |  |  |  |  |  |  |  | *twk-8* | TWiK family of potassium channels |
|  |  |  |  |  |  |  |  |  |  |  |  |  |  |  |  |  |  |  |  |  |  |  |  |  |  |  |  |  |  |  |  |  |  |  |  |  |  | Y97E10AR.2 |  |
|  |  |  |  |  |  |  |  |  |  |  |  |  |  |  |  |  |  |  |  |  |  |  |  |  |  |  |  |  |  |  |  |  |  |  |  |  |  | Y69E1A.5 |  |
|  |  |  |  |  |  |  |  |  |  |  |  |  |  |  |  |  |  |  |  |  |  |  |  |  |  |  |  |  |  |  |  |  |  |  |  |  |  | *unc-27* | UNCoordinated |
|  |  |  |  |  |  |  |  |  |  |  |  |  |  |  |  |  |  |  |  |  |  |  |  |  |  |  |  |  |  |  |  |  |  |  |  |  |  | *hsp-12.1* | Heat Shock Protein |
|  |  |  |  |  |  |  |  |  |  |  |  |  |  |  |  |  |  |  |  |  |  |  |  |  |  |  |  |  |  |  |  |  |  |  |  |  |  | *heh-1* | HE1 homologue |
|  |  |  |  |  |  |  |  |  |  |  |  |  |  |  |  |  |  |  |  |  |  |  |  |  |  |  |  |  |  |  |  |  |  |  |  |  |  | *ttr-36* | TransThyretin-Related family domain |
|  |  |  |  |  |  |  |  |  |  |  |  |  |  |  |  |  |  |  |  |  |  |  |  |  |  |  |  |  |  |  |  |  |  |  |  |  |  | *ttr-35* | TransThyretin-Related family domain |
|  |  |  |  |  |  |  |  |  |  |  |  |  |  |  |  |  |  |  |  |  |  |  |  |  |  |  |  |  |  |  |  |  |  |  |  |  |  | C18B2.3 |  |
|  |  |  |  |  |  |  |  |  |  |  |  |  |  |  |  |  |  |  |  |  |  |  |  |  |  |  |  |  |  |  |  |  |  |  |  |  |  | T04B2.3 |  |
|  |  |  |  |  |  |  |  |  |  |  |  |  |  |  |  |  |  |  |  |  |  |  |  |  |  |  |  |  |  |  |  |  |  |  |  |  |  | D2063.1 |  |
|  |  |  |  |  |  |  |  |  |  |  |  |  |  |  |  |  |  |  |  |  |  |  |  |  |  |  |  |  |  |  |  |  |  |  |  |  |  | ZK1058.9 |  |
|  |  |  |  |  |  |  |  |  |  |  |  |  |  |  |  |  |  |  |  |  |  |  |  |  |  |  |  |  |  |  |  |  |  |  |  |  |  | *ptr-12* | PaTched Related family |
|  |  |  |  |  |  |  |  |  |  |  |  |  |  |  |  |  |  |  |  |  |  |  |  |  |  |  |  |  |  |  |  |  |  |  |  |  |  | R04B5.6 |  |
|  |  |  |  |  |  |  |  |  |  |  |  |  |  |  |  |  |  |  |  |  |  |  |  |  |  |  |  |  |  |  |  |  |  |  |  |  |  | F21D9.4 |  |
|  |  |  |  |  |  |  |  |  |  |  |  |  |  |  |  |  |  |  |  |  |  |  |  |  |  |  |  |  |  |  |  |  |  |  |  |  |  | F13H10.5 |  |
|  |  |  |  |  |  |  |  |  |  |  |  |  |  |  |  |  |  |  |  |  |  |  |  |  |  |  |  |  |  |  |  |  |  |  |  |  |  | C48E7.6 |  |
|  |  |  |  |  |  |  |  |  |  |  |  |  |  |  |  |  |  |  |  |  |  |  |  |  |  |  |  |  |  |  |  |  |  |  |  |  |  | *clhm-1* | CaLcium Homeostasis Modulator |
|  |  |  |  |  |  |  |  |  |  |  |  |  |  |  |  |  |  |  |  |  |  |  |  |  |  |  |  |  |  |  |  |  |  |  |  |  |  | *unc-68* | UNCoordinated |
|  |  |  |  |  |  |  |  |  |  |  |  |  |  |  |  |  |  |  |  |  |  |  |  |  |  |  |  |  |  |  |  |  |  |  |  |  |  | *tmd-2* | TropoMoDulin |
|  |  |  |  |  |  |  |  |  |  |  |  |  |  |  |  |  |  |  |  |  |  |  |  |  |  |  |  |  |  |  |  |  |  |  |  |  |  | F20D6.10 |  |
|  |  |  |  |  |  |  |  |  |  |  |  |  |  |  |  |  |  |  |  |  |  |  |  |  |  |  |  |  |  |  |  |  |  |  |  |  |  | *rrc-1* | RhoGAP for Rac-1 and Cdc-42 |
|  |  |  |  |  |  |  |  |  |  |  |  |  |  |  |  |  |  |  |  |  |  |  |  |  |  |  |  |  |  |  |  |  |  |  |  |  |  | K09A9.6 |  |
|  |  |  |  |  |  |  |  |  |  |  |  |  |  |  |  |  |  |  |  |  |  |  |  |  |  |  |  |  |  |  |  |  |  |  |  |  |  | F54E2.1 |  |
|  |  |  |  |  |  |  |  |  |  |  |  |  |  |  |  |  |  |  |  |  |  |  |  |  |  |  |  |  |  |  |  |  |  |  |  |  |  | C07D10.1 |  |
|  |  |  |  |  |  |  |  |  |  |  |  |  |  |  |  |  |  |  |  |  |  |  |  |  |  |  |  |  |  |  |  |  |  |  |  |  |  | *sup-10* | SUPpressor |
|  |  |  |  |  |  |  |  |  |  |  |  |  |  |  |  |  |  |  |  |  |  |  |  |  |  |  |  |  |  |  |  |  |  |  |  |  |  | *nhr-19* | Nuclear Hormone Receptor family |
|  |  |  |  |  |  |  |  |  |  |  |  |  |  |  |  |  |  |  |  |  |  |  |  |  |  |  |  |  |  |  |  |  |  |  |  |  |  | Y73E7A.3 |  |
|  |  |  |  |  |  |  |  |  |  |  |  |  |  |  |  |  |  |  |  |  |  |  |  |  |  |  |  |  |  |  |  |  |  |  |  |  |  | T01B4.3 |  |
|  |  |  |  |  |  |  |  |  |  |  |  |  |  |  |  |  |  |  |  |  |  |  |  |  |  |  |  |  |  |  |  |  |  |  |  |  |  | F15B10.3 |  |
|  |  |  |  |  |  |  |  |  |  |  |  |  |  |  |  |  |  |  |  |  |  |  |  |  |  |  |  |  |  |  |  |  |  |  |  |  |  | *gstk-2* | Glutathione S-Transferase Kappa protein |
|  |  |  |  |  |  |  |  |  |  |  |  |  |  |  |  |  |  |  |  |  |  |  |  |  |  |  |  |  |  |  |  |  |  |  |  |  |  | F53F10.1 |  |
|  |  |  |  |  |  |  |  |  |  |  |  |  |  |  |  |  |  |  |  |  |  |  |  |  |  |  |  |  |  |  |  |  |  |  |  |  |  | *far-6* | Fatty Acid/Retinol binding protein |
|  |  |  |  |  |  |  |  |  |  |  |  |  |  |  |  |  |  |  |  |  |  |  |  |  |  |  |  |  |  |  |  |  |  |  |  |  |  | *linc-13* | Long Intervening Non-Coding RNA |
|  |  |  |  |  |  |  |  |  |  |  |  |  |  |  |  |  |  |  |  |  |  |  |  |  |  |  |  |  |  |  |  |  |  |  |  |  |  | *ins-27* | INSulin related |
|  |  |  |  |  |  |  |  |  |  |  |  |  |  |  |  |  |  |  |  |  |  |  |  |  |  |  |  |  |  |  |  |  |  |  |  |  |  | C06A1.2 |  |
|  |  |  |  |  |  |  |  |  |  |  |  |  |  |  |  |  |  |  |  |  |  |  |  |  |  |  |  |  |  |  |  |  |  |  |  |  |  | F48F5.6 |  |
|  |  |  |  |  |  |  |  |  |  |  |  |  |  |  |  |  |  |  |  |  |  |  |  |  |  |  |  |  |  |  |  |  |  |  |  |  |  | Y71H2B.4 |  |
|  |  |  |  |  |  |  |  |  |  |  |  |  |  |  |  |  |  |  |  |  |  |  |  |  |  |  |  |  |  |  |  |  |  |  |  |  |  | K02D7.2 |  |
|  |  |  |  |  |  |  |  |  |  |  |  |  |  |  |  |  |  |  |  |  |  |  |  |  |  |  |  |  |  |  |  |  |  |  |  |  |  | F27C8.2 |  |
|  |  |  |  |  |  |  |  |  |  |  |  |  |  |  |  |  |  |  |  |  |  |  |  |  |  |  |  |  |  |  |  |  |  |  |  |  |  | M7.12 |  |
|  |  |  |  |  |  |  |  |  |  |  |  |  |  |  |  |  |  |  |  |  |  |  |  |  |  |  |  |  |  |  |  |  |  |  |  |  |  | B0495.17 |  |
|  |  |  |  |  |  |  |  |  |  |  |  |  |  |  |  |  |  |  |  |  |  |  |  |  |  |  |  |  |  |  |  |  |  |  |  |  |  | C33B4.4 |  |
|  |  |  |  |  |  |  |  |  |  |  |  |  |  |  |  |  |  |  |  |  |  |  |  |  |  |  |  |  |  |  |  |  |  |  |  |  |  | K08C7.7 |  |
|  |  |  |  |  |  |  |  |  |  |  |  |  |  |  |  |  |  |  |  |  |  |  |  |  |  |  |  |  |  |  |  |  |  |  |  |  |  | Y57G11C.40 |  |
|  |  |  |  |  |  |  |  |  |  |  |  |  |  |  |  |  |  |  |  |  |  |  |  |  |  |  |  |  |  |  |  |  |  |  |  |  |  | Y71H2AL.2 |  |
|  |  |  |  |  |  |  |  |  |  |  |  |  |  |  |  |  |  |  |  |  |  |  |  |  |  |  |  |  |  |  |  |  |  |  |  |  |  | Y71F9AR.2 |  |
|  |  |  |  |  |  |  |  |  |  |  |  |  |  |  |  |  |  |  |  |  |  |  |  |  |  |  |  |  |  |  |  |  |  |  |  |  |  | F21H7.3 |  |

### Phenotypes enriched

none found

### Anatomy terms enriched

|  |  |  |  |
| --- | --- | --- | --- |
| **Group name** | **Number in cluster** | **Enrichment** | **FDR corrected p** |
| body wall musculature | 46 | 3.43 | 1.04e-09 |
| muscular system | 50 | 2.54 | 1.35e-06 |
| vulval muscle | 23 | 3.22 | 1.92e-03 |
| body muscle cell | 26 | 2.43 | 4.02e-02 |

### GO terms enriched

|  |  |  |
| --- | --- | --- |
| **GO term** | **Number of genes** | **FDR-corrected p-value** |
| contractile fiber | 13 | 1.2e-06 |
| extracellular region | 16 | 3.2e-04 |
| extracellular space | 8 | 1.2e-03 |
| striated muscle dense body | 7 | 2.9e-03 |
| actin filament-based process | 8 | 2.8e-02 |

### Expression clusters enriched

|  |  |  |  |
| --- | --- | --- | --- |
| **Group name** | **Number in cluster** | **Enrichment** | **FDR corrected p** |
| Genes enriched in muscle cells (0hr muscle dataset). Dissociated myo-3::GFP embryos were cultured for 0 hours before FACS sorting. | 129 | 13.83 | 1.47e-119 |
| Total muscle enriched genes (complete list of non-overlapping genes from the 0hr and 24hr muscle enriched datasets). | 137 | 8.70 | 5.04e-101 |
| Genes enriched in muscle cells (24hr muscle dataset). Dissociated myo-3::GFP embryos were cultured for 24 hours before FACS sorting. | 102 | 9.08 | 2.51e-70 |
| Genes that show selective expression in a subset of cell types vs broadly expressed in many cell types. Correspond to 20% - 57% of enriched\_genes for a given cell type. WBPaper00037950:bodywall-muscle\_larva\_SelectivelyEnriched | 71 | 12.95 | 1.79e-56 |
| Genes enriched in HLH-1 heat shock dataset. | 117 | 4.12 | 1.51e-45 |
| Genes that show selective expression in a subset of cell types vs broadly expressed in many cell types. Correspond to 20% - 57% of enriched\_genes for a given cell type. WBPaper00037950:bodywall-muscle\_embryo\_SelectivelyEnriched | 46 | 20.76 | 1.37e-44 |
| Genes significantly enriched (> 2x, FDR < 5%) in a particular cell-type versus a reference sample of all cells at both embryonic and larval stages. WBPaper00037950:bodywall-muscle\_CoreEnriched | 36 | 24.30 | 6.67e-37 |
| Genes significantly enriched (> 2x, FDR < 5%) in a particular cell-type versus a reference sample of all cells at the same stage. WBPaper00037950:bodywall-muscle\_embryo\_enriched | 52 | 10.78 | 1.24e-35 |
| Genes significantly enriched (> 2x, FDR < 5%) in a particular cell-type versus a reference sample of all cells at the same stage. WBPaper00037950:bodywall-muscle\_larva\_enriched | 78 | 5.55 | 3.70e-35 |
| Genes enriched in body wall muscle AIN-2 miRISCs. Pmyo-3-ain-2-gfp IP was performed in mixed stage worms. | 41 | 6.40 | 1.17e-18 |
| Genes predicted to be downregulated more than 2.0 fold in (AFD+AWB) datasets as compared to unsorted whole embryonic cells dataset. | 35 | 5.44 | 2.11e-13 |
| Genes with expression level down regulated in mir-35 mutants comparing with N2. | 47 | 3.79 | 9.93e-13 |
| WT-Pico Pan-neural Depleted Genes, with genes found multiple times in a single dataset removed (without dups). | 43 | 2.79 | 2.93e-07 |
| TGF- Dauer pathway adult transcriptional targets. Results obtained by comparing the microarray results of the dauer-constitutive mutants daf-7(e1372), daf-7(m62), and daf-1(m40) with dauer-defective mutants daf-3(mgDf90), daf-5(e1386), and daf-7(e1372);daf-3(mgDf90) double mutants at the permissive temperature, 20C, on the first day of adulthood. WBPaper00031040:TGF-beta\_adult\_upregulated | 58 | 2.18 | 1.81e-06 |
| Genes with no change in hcf-1(-), no change in sir-2.1(O/E) and upregulated in daf-2(-). | 34 | 3.06 | 2.51e-06 |
| Genes that showed increased expression after treated with 2-deoxy-D-glucose. | 17 | 5.49 | 7.45e-06 |
| Genes downregulated by CYD-1/CDK-4 for more than two fold. | 23 | 3.48 | 8.61e-05 |
| The cluster contains genes that are significantly enriched in L1 muscle. | 40 | 2.26 | 2.71e-04 |
| Expression Pattern Group C, enriched for genes involved in metabolic processes. | 31 | 2.55 | 4.94e-04 |
| Genes differentially expressed under UVC exposure and EtBr treatment vs under EtBr treatment but without UVC exposure at the -3h timepoint (3 h after the third UVC dose (51h), which is also 3 h after being placed on food). | 38 | 2.07 | 3.43e-03 |
| Developmentally modulated gene cluster. cgc4386\_cluster\_1\_5 | 9 | 5.07 | 1.84e-02 |
| Differentially expressed genes during worm lifespan. Medoid 2 Fig.4. | 26 | 2.24 | 2.26e-02 |
| Genes differentially expressed in control vs after UVC exposure and EtBr treatment at the 3h timepoint (3 h after the third UVC dose (51h), which is also 3 h after being placed on food). | 70 | 1.49 | 3.04e-02 |
| Genes with expression level down in zfp-1 mutant background. | 14 | 3.08 | 4.31e-02 |
| Genes that are dosage compensated. | 14 | 3.06 | 4.61e-02 |
| Germline-enriched and sex-biased expression profile cluster A. | 19 | 2.50 | 4.87e-02 |

### Motifs enriched

|  |  |  |  |  |  |
| --- | --- | --- | --- | --- | --- |
| **Motif** | **Logo** | **Possible orthologs** | **Number of motifs in cluster** | **Enrichment** | **FDR corrected p** |
| pTH10808 |  | ztf-19 | 61 | 3.74 | 2.5e-16 |
| MA0545.1 |  | hlh-1 (0.87) hlh-11 lin-32 hlh-15 hlh-14 | 61 | 3.55 | 2.8e-15 |
| Myf6\_3824 |  | hlh-1 (0.87) K02D7.2 (0.79) hlh-8 (0.76) hlh-15 hlh-2 ces-1 | 59 | 3.00 | 1.1e-11 |
| pTH6071 |  | C46E10.8 C33G8.2 | 82 | 2.24 | 9.0e-11 |
| Egr1\_2580 |  | ZC328.2 | 97 | 1.91 | 1.5e-09 |
| MA0543.1 |  | eor-1 daf-8 | 108 | 1.78 | 1.9e-09 |
| pTH9907 |  | nhr-34 | 119 | 1.66 | 5.0e-09 |
| pTH9924 |  | nhr-46 | 95 | 1.87 | 8.9e-09 |
| Mw156 |  | hlh-30 pax-1 aha-1 mdl-1 | 106 | 1.75 | 9.3e-09 |
| pTH9163 |  | nhr-3 | 127 | 1.57 | 1.5e-08 |
| Oli\_da\_SANGER\_5\_3\_FBgn0032651 |  | hlh-8 (0.76) hlh-32 hlh-15 ngn-1 hlh-12 | 86 | 1.96 | 1.6e-08 |
| pTH9354 |  | ZC328.2 daf-16 | 87 | 1.94 | 1.7e-08 |
| Caup\_Cell\_FBgn0015919 |  | fkh-9 daf-16 irx-1 | 126 | 1.56 | 3.4e-08 |
| pTH9340 |  | mab-9 tbx-39 tbx-38 tbx-42 tbx-43 | 102 | 1.75 | 3.4e-08 |
| MYB\_f1 |  | D1081.8 | 62 | 2.35 | 3.5e-08 |
| pTH9142 |  | ztf-6 C34D1.1 gei-11 | 93 | 1.84 | 3.5e-08 |
| MEIS2\_do |  | ceh-32 lin-39 lin-32 | 53 | 2.59 | 4.4e-08 |
| MA0599.1 |  | ZC328.2 klf-1 klf-2 | 117 | 1.61 | 6.4e-08 |
| pTH10638 |  | dmd-3 C34D1.1 | 100 | 1.74 | 9.0e-08 |
| EGR1\_1 |  | ZC328.2 klf-1 klf-2 | 107 | 1.68 | 9.5e-08 |
| MITF\_f1 |  | hlh-30 irx-1 mxl-1 | 99 | 1.75 | 1.0e-07 |
| pTH1001 |  | dnj-17 | 107 | 1.68 | 1.0e-07 |
| V$GATA1\_06 |  | elt-1 ztf-29 | 113 | 1.62 | 1.5e-07 |
| V$GR\_Q6 |  | nhr-255 | 121 | 1.56 | 1.6e-07 |
| Nr2f6\_2 |  | nhr-19 (0.8) nhr-2 nhr-62 | 95 | 1.75 | 3.1e-07 |
| tll\_FlyReg\_FBgn0003720 |  | nhr-2 nhr-239 nhr-62 | 86 | 1.84 | 3.6e-07 |
| pTH2818 |  | eor-1 ref-2 egrh-3 | 89 | 1.80 | 3.8e-07 |
| MA0204.1 |  | ceh-32 dmd-4 dmd-5 | 98 | 1.71 | 4.0e-07 |
| MA0537.1 |  | blmp-1 | 97 | 1.72 | 4.1e-07 |
| Tcf7\_0950 |  | pop-1 | 93 | 1.76 | 4.3e-07 |
| PLAG1\_si |  | Y53H1A.2 klf-2 plp-2 | 62 | 2.19 | 4.3e-07 |
| Tin\_SOLEXA\_FBgn0004110 |  | dsc-1 (0.72) ceh-22 ceh-24 | 102 | 1.67 | 4.4e-07 |
| CG31670\_SOLEXA\_5\_FBgn0031375 |  | F21A9.2 CELE\_Y38H8A.5 | 114 | 1.58 | 4.4e-07 |
| Mw154 |  | lin-39 ceh-20 ceh-12 | 74 | 1.98 | 4.5e-07 |
| Nkx2-3\_3435 |  | dsc-1 (0.72) ceh-24 | 99 | 1.70 | 4.8e-07 |
| HeLa-S3\_RFX5\_Stanford |  | daf-19 | 109 | 1.62 | 4.8e-07 |
| pTH10645 |  | nhr-100 nhr-10 nhr-7 elt-1 | 97 | 1.71 | 5.0e-07 |
| pTH3751 |  | tbx-39 | 113 | 1.59 | 5.2e-07 |
| pTH5119 |  | cfi-1 | 103 | 1.66 | 5.4e-07 |
| ELF2\_f1 |  | lin-1 C24A1.2 | 124 | 1.51 | 5.5e-07 |
| foxo\_SANGER\_10\_FBgn0038197 |  | lin-31 (0.67) let-381 (0.64) fkh-8 fkh-7 fkh-10 daf-16 | 93 | 1.75 | 5.5e-07 |
| NEUROG2\_2 |  | hlh-8 (0.76) lin-31 (0.67) hlh-32 hlh-15 ngn-1 | 97 | 1.71 | 5.8e-07 |
| pTH6003 |  | nhr-182 nhr-134 | 112 | 1.59 | 6.0e-07 |
| Prop1\_3949 |  | ceh-53 (-0.56) ceh-16 | 174 | 1.24 | 6.6e-07 |
| CG7386\_F10-12\_SANGER\_5\_FBgn0035691 |  | F56D1.1 gei-3 sox-4 | 120 | 1.53 | 6.8e-07 |
| HXD9\_f1 |  | php-3 lin-39 hbl-1 | 91 | 1.76 | 6.8e-07 |
| pTH9182 |  | tbx-39 | 107 | 1.61 | 8.8e-07 |
| GABPA\_1 |  | lin-1 C24A1.2 | 109 | 1.59 | 1.1e-06 |
| TCF7L1\_1 |  | nhr-100 pop-1 sox-4 | 133 | 1.44 | 1.3e-06 |
| pTH9244 |  | tbx-39 | 98 | 1.67 | 1.3e-06 |
| SOX2\_5 |  | dmd-4 grh-1 sox-4 | 131 | 1.45 | 1.4e-06 |
| GATA3\_si |  | elt-1 | 44 | 2.61 | 1.5e-06 |
| Foxj3\_2 |  | lin-31 (0.67) let-381 (0.64) fkh-8 pha-4 fkh-7 fkh-10 daf-16 | 101 | 1.64 | 1.6e-06 |
| pTH3998 |  | tbx-39 | 100 | 1.65 | 1.7e-06 |
| pTH10811 |  | nhr-216 nhr-142 nhr-84 | 125 | 1.48 | 1.8e-06 |
| Hoxc9\_2367 |  | lin-39 pal-1 ceh-18 alr-1 ceh-12 | 122 | 1.49 | 1.9e-06 |
| NK7.1\_Cell\_FBgn0024321 |  | cog-1 (0.53) ceh-1 ceh-43 lin-39 ceh-30 ceh-19 lim-7 alr-1 ceh-8 ceh-9 ceh-31 ceh-24 | 140 | 1.40 | 1.9e-06 |
| pTH9215 |  | C34D1.1 | 109 | 1.58 | 2.0e-06 |
| Caup\_SOLEXA\_FBgn0015919 |  | irx-1 | 108 | 1.58 | 2.2e-06 |
| SP3\_1 |  | klf-1 klf-2 | 99 | 1.65 | 2.2e-06 |
| Gata3\_1024 |  | elt-1 | 112 | 1.55 | 2.3e-06 |
| Tcf7l2\_3461 |  | ceh-20 pop-1 | 116 | 1.52 | 2.4e-06 |
| SPDEF\_3 |  | nhr-100 lin-39 lin-1 | 87 | 1.75 | 2.4e-06 |
| pTH5169 |  | cfi-1 | 98 | 1.65 | 2.5e-06 |
| pTH9246 |  | lin-31 (0.67) let-381 (0.64) fkh-10 C34D1.1 | 94 | 1.69 | 2.5e-06 |
| pTH10816 |  | dmd-6 | 141 | 1.39 | 2.5e-06 |
| MA0547.1 |  | ceh-2 skn-1 | 118 | 1.51 | 2.7e-06 |
| pTH5437 |  | ceh-34 | 81 | 1.81 | 2.8e-06 |
| Elk4 |  | lin-1 F19F10.1 C24A1.2 | 112 | 1.54 | 3.0e-06 |
| pTH9394 |  | klf-1 klf-2 sptf-3 | 99 | 1.64 | 3.1e-06 |
| V$HLF\_01 |  | atf-2 ces-2 C48E7.11 | 111 | 1.55 | 3.1e-06 |
| pTH8991 |  | cey-3 | 102 | 1.61 | 3.5e-06 |
| NR4A2\_si |  | nhr-6 nhr-2 nhr-10 nhr-213 nhr-71 nhr-68 Y67D8A.3 | 111 | 1.55 | 3.5e-06 |
| pTH5891 |  | nhr-49 | 86 | 1.74 | 3.6e-06 |
| Hoxc10\_2779 |  | cfi-1 php-3 lin-39 unc-86 lim-7 | 118 | 1.50 | 3.6e-06 |
| MA0542.1 |  | elt-3 (0.62) elt-7 (-0.57) ceh-32 elt-6 elt-1 egl-27 ceh-34 | 73 | 1.89 | 3.7e-06 |
| pTH9708 |  | ceh-34 | 91 | 1.70 | 3.7e-06 |
| PBDE\_GATA1\_UCD |  | elt-1 | 113 | 1.53 | 3.9e-06 |
| pTH9085 |  | nhr-42 | 95 | 1.66 | 4.0e-06 |
| Six6\_2267 |  | ceh-32 ceh-34 | 68 | 1.96 | 4.2e-06 |
| MA0126.1 |  | dmd-3 pax-3 lin-48 | 72 | 1.90 | 4.3e-06 |
| V$FOXJ2\_02 |  | lin-31 (0.67) elt-1 | 81 | 1.79 | 4.4e-06 |
| pTH9384 |  | cfi-1 | 99 | 1.62 | 4.5e-06 |
| Tcf3\_3787 |  | pop-1 | 120 | 1.48 | 4.8e-06 |
| CG4328\_Cell\_FBgn0036274 |  | dsc-1 (0.72) egl-5 (0.57) ceh-53 (-0.56) cog-1 (0.53) ceh-1 ceh-43 ceh-45 lin-39 pha-2 pal-1 ceh-30 ceh-36 ceh-14 lim-7 ceh-18 alr-1 ceh-2 ceh-12 lim-4 lim-6 and 9 others  [full list] | 98 | 1.63 | 5.2e-06 |
| GSE15244\_FoxA1 |  | lin-31 (0.67) let-381 (0.64) | 104 | 1.58 | 5.3e-06 |
| pTH5690 |  | ceh-32 | 91 | 1.68 | 5.9e-06 |
| pTH9049 |  | ztf-6 ztf-2 | 123 | 1.46 | 6.0e-06 |
| HOXC10\_2 |  | php-3 lin-39 pal-1 ceh-24 | 133 | 1.41 | 6.0e-06 |
| V$E47\_02 |  | lin-32 hlh-2 | 55 | 2.17 | 6.2e-06 |
| Six3\_1732 |  | ceh-34 | 85 | 1.73 | 6.8e-06 |
| NR5A2\_f1 |  | nhr-68 | 96 | 1.63 | 7.0e-06 |
| MA0151.1 |  | cfi-1 ceh-14 alr-1 ZC204.2 | 118 | 1.49 | 7.0e-06 |
| EHF\_si |  | nhr-19 (0.8) lin-1 C24A1.2 | 111 | 1.53 | 7.2e-06 |
| Sox1\_2631 |  | sox-4 | 114 | 1.51 | 7.6e-06 |
| TBX3\_f1 |  | ceh-45 tbx-39 | 135 | 1.39 | 8.0e-06 |
| Mw144 |  | elt-1 | 116 | 1.49 | 8.0e-06 |
| Sox30\_2781 |  | gei-3 pop-1 sox-4 C05C9.3 | 91 | 1.67 | 8.0e-06 |
| pTH10795 |  | ztf-14 ZC328.2 lin-1 ref-2 | 88 | 1.69 | 8.3e-06 |
| MA0007.2 |  | nhr-255 npax-1 lin-14 | 113 | 1.51 | 8.4e-06 |
| pTH9300 |  | dmd-3 C34D1.1 | 115 | 1.50 | 9.1e-06 |
| pTH9125 |  | K11D2.4 egl-13 sox-4 | 97 | 1.61 | 9.8e-06 |
| OTX2\_si |  | ceh-53 (-0.56) ceh-45 pha-2 ceh-36 alr-1 dve-1 | 110 | 1.52 | 1.1e-05 |
| CG2052\_SANGER\_2.5\_FBgn0039905 |  | fkh-7 mel-28 lin-29 | 110 | 1.52 | 1.1e-05 |
| pTH9198 |  | dmd-3 | 106 | 1.55 | 1.1e-05 |
| Sox17\_2837 |  | sox-4 | 136 | 1.38 | 1.1e-05 |
| ZN384\_f1 |  | K11D2.4 lin-29 | 65 | 1.94 | 1.2e-05 |
| ETS2\_f1 |  | lin-1 C24A1.2 | 123 | 1.44 | 1.2e-05 |
| NHLH1\_2 |  | hlh-1 (0.87) hlh-15 | 61 | 2.00 | 1.4e-05 |
| V$ARNT\_02 |  | hlh-30 lin-22 hlh-26 aha-1 mxl-1 mxl-2 C27D6.4 | 75 | 1.80 | 1.5e-05 |
| CEBPE\_f1 |  | C48E7.11 R07H5.10 | 85 | 1.69 | 1.6e-05 |
| HXD10\_f1 |  | php-3 nhr-2 | 126 | 1.42 | 1.7e-05 |
| FOXD3\_1 |  | lin-31 (0.67) let-381 (0.64) ceh-53 (-0.56) ceh-43 hmg-12 hmbx-1 Y116A8C.22 | 57 | 2.06 | 1.8e-05 |
| IRX2\_1 |  | irx-1 | 101 | 1.57 | 1.8e-05 |
| MA0503.1 |  | pzf-1 (-0.64) ceh-24 | 104 | 1.55 | 1.8e-05 |
| Six1\_0935 |  | ceh-32 | 83 | 1.71 | 1.8e-05 |
| pTH9969 |  | pag-3 | 154 | 1.29 | 2.0e-05 |
| V$FAC1\_01 |  | gei-8 | 130 | 1.40 | 2.0e-05 |
| Spdef\_0905 |  | lin-1 | 94 | 1.61 | 2.1e-05 |
| V$CDPCR3\_01 |  | ceh-48 ceh-24 | 106 | 1.53 | 2.1e-05 |
| Cdx2\_4272 |  | ceh-13 | 117 | 1.46 | 2.2e-05 |
| pTH10718 |  | egl-43 | 109 | 1.51 | 2.2e-05 |
| V$MYB\_Q6 |  | D1081.8 | 95 | 1.60 | 2.4e-05 |
| Lmx1a\_2238 |  | lim-7 lim-6 ceh-24 ceh-16 | 111 | 1.49 | 2.4e-05 |
| pTH10623 |  | scrt-1 | 105 | 1.53 | 2.5e-05 |
| Bbx\_3753 |  | cfi-1 lim-6 nhr-208 gei-3 | 107 | 1.52 | 2.6e-05 |
| SMAD3\_f1 |  | daf-8 | 86 | 1.66 | 2.7e-05 |
| HepG2\_HSF1\_Stanford |  | Y53C10A.3 | 95 | 1.59 | 2.7e-05 |
| pTH10837 |  | ceh-24 ces-1 T22H9.4 C34H4.5 | 115 | 1.47 | 2.8e-05 |
| ONECUT2\_1 |  | dsc-1 (0.72) ceh-48 | 128 | 1.40 | 3.2e-05 |
| pTH6327 |  | dsc-1 (0.72) | 96 | 1.58 | 3.3e-05 |
| So\_Cell\_FBgn0003460 |  | ceh-32 ztf-28 skn-1 | 79 | 1.72 | 3.5e-05 |
| MCR\_f1 |  | nhr-255 | 99 | 1.56 | 3.6e-05 |
| Hnf4a\_2640 |  | nhr-62 | 84 | 1.67 | 3.8e-05 |
| ZNF75A\_1 |  | ztf-3 lag-1 | 128 | 1.39 | 3.9e-05 |
| HXD13\_f1 |  | pal-1 mex-6 | 126 | 1.40 | 4.0e-05 |
| Sox15\_3457 |  | sox-4 | 121 | 1.42 | 4.3e-05 |
| MA0150.2 |  | sknr-1 jun-1 fos-1 crh-1 F45H11.6 | 67 | 1.84 | 4.5e-05 |
| MA0102.3 |  | C48E7.11 | 76 | 1.73 | 4.7e-05 |
| I$SN\_02 |  | K02D7.2 (0.79) C24A1.2 | 103 | 1.52 | 4.8e-05 |
| pTH5561 |  | nhr-239 | 105 | 1.51 | 5.0e-05 |
| pTH6591 |  | lin-31 (0.67) | 101 | 1.53 | 5.2e-05 |
| Msx1\_3031 |  | ceh-53 (-0.56) ceh-1 ceh-43 ceh-45 lin-39 npax-3 ceh-18 alr-1 ceh-12 | 126 | 1.39 | 5.2e-05 |
| pTH6508 |  | nhr-36 | 112 | 1.46 | 5.4e-05 |
| SRP000217\_Sox2 |  | ceh-18 ceh-6 sox-4 | 131 | 1.37 | 5.8e-05 |
| Hoxc8\_3429 |  | lin-39 | 103 | 1.51 | 6.0e-05 |
| MA0452.2 |  | B0310.2 (0.54) ZK177.3 | 71 | 1.77 | 6.2e-05 |
| MA0038.1 |  | odd-1 lim-6 | 75 | 1.73 | 6.5e-05 |
| Cdx1\_2245 |  | ceh-13 | 67 | 1.82 | 6.6e-05 |
| Exd\_SOLEXA\_FBgn0000611 |  | ceh-32 ceh-20 ces-1 F55C5.11 | 121 | 1.41 | 6.7e-05 |
| Hoxa5\_3415 |  | lin-39 | 97 | 1.55 | 6.7e-05 |
| pTH10823 |  | B0310.2 (0.54) | 96 | 1.55 | 6.8e-05 |
| V$FREAC7\_01 |  | lin-31 (0.67) | 86 | 1.62 | 7.5e-05 |
| CG4854\_SANGER\_10\_FBgn0038766 |  | K11D2.4 mxl-1 | 145 | 1.30 | 7.5e-05 |
| retn\_SANGER\_5\_FBgn0004795 |  | let-381 (0.64) cfi-1 ceh-20 | 96 | 1.55 | 7.7e-05 |
| pTH5118 |  | cfi-1 | 62 | 1.88 | 7.8e-05 |
| BARHL2\_2 |  | ceh-1 ceh-43 lin-39 lim-7 alr-1 ceh-9 ceh-31 ceh-16 | 55 | 1.99 | 7.8e-05 |
| pTH6486 |  | nhr-145 | 80 | 1.67 | 7.9e-05 |
| SRP001585\_Tbx2 |  | lin-1 tbx-39 odr-7 nhr-28 slr-2 nhr-273 | 100 | 1.52 | 8.4e-05 |
| pTH9262 |  | lin-54 | 123 | 1.40 | 8.5e-05 |
| pTH6423 |  | pha-2 | 98 | 1.53 | 8.7e-05 |
| pTH10629 |  | ztf-3 hif-1 daf-12 ceh-9 Y5F2A.4 | 102 | 1.51 | 8.8e-05 |
| pTH9237 |  | mel-28 | 84 | 1.63 | 9.4e-05 |
| Elf3 |  | C24A1.2 | 125 | 1.39 | 9.4e-05 |
| V$AREB6\_02 |  | ztf-6 | 89 | 1.59 | 9.6e-05 |
| pTH9925 |  | nhr-100 ztf-11 | 132 | 1.35 | 9.8e-05 |
| Prrx1\_3442 |  | ceh-1 ceh-45 lin-39 eyg-1 alr-1 ceh-10 | 178 | 1.17 | 9.9e-05 |
| HLH27 |  | hlh-27 hlh-28 | 88 | 1.60 | 1.0e-04 |
| pTH5928 |  | ceh-34 | 82 | 1.64 | 1.0e-04 |
| Pou2f2\_3748 |  | lim-7 ceh-18 alr-1 | 107 | 1.47 | 1.0e-04 |
| MA0066.1 |  | nhr-43 (0.53) nhr-6 nhr-71 | 88 | 1.59 | 1.1e-04 |
| Hoxa13\_3126 |  | pal-1 ceh-24 | 104 | 1.49 | 1.1e-04 |
| Spdef |  | lin-1 | 99 | 1.52 | 1.2e-04 |
| Hoxd8\_2644 |  | lin-39 ceh-18 ceh-6 | 99 | 1.51 | 1.2e-04 |
| HOXC10\_3 |  | php-3 lin-39 pal-1 ceh-13 ceh-24 D1005.3 T27F2.4 | 112 | 1.44 | 1.2e-04 |
| pTH4269 |  | nhr-2 F13H6.1 nhr-86 nhr-177 | 97 | 1.53 | 1.2e-04 |
| pTH9709 |  | die-1 | 116 | 1.42 | 1.3e-04 |
| Tbp\_pr781 |  | tbp-1 | 74 | 1.70 | 1.4e-04 |
| Lhx1\_2240 |  | lim-7 | 179 | 1.16 | 1.4e-04 |
| Nkx1-1\_3856 |  | ceh-30 | 161 | 1.23 | 1.5e-04 |
| pTH8998 |  | mab-3 daf-19 | 113 | 1.43 | 1.5e-04 |
| MA0173.1 |  | D1081.8 hlh-32 irx-1 | 111 | 1.44 | 1.6e-04 |
| pTH5257 |  | C48E7.11 | 70 | 1.74 | 1.6e-04 |
| pTH10777 |  | dmd-3 | 144 | 1.29 | 1.7e-04 |
| pTH10647 |  | nhr-232 | 103 | 1.48 | 1.7e-04 |
| Vsx1\_1728 |  | alr-1 | 175 | 1.17 | 1.7e-04 |
| pTH9242 |  | mel-28 | 107 | 1.46 | 1.7e-04 |
| Dlx3\_1030 |  | ceh-43 | 176 | 1.17 | 1.8e-04 |
| pTH6447 |  | ceh-19 | 65 | 1.78 | 1.9e-04 |
| MA0386.1 |  | che-1 (0.52) K11D2.4 tbp-1 | 108 | 1.45 | 2.0e-04 |
| Vax1\_3499 |  | C02F12.10 | 96 | 1.51 | 2.1e-04 |
| KLF8\_f1 |  | ZC328.2 klf-1 | 88 | 1.57 | 2.1e-04 |
| GM12878\_PBX3\_HudsonAlpha |  | lin-31 (0.67) nfya-2 ceh-20 cey-3 dro-1 | 97 | 1.51 | 2.2e-04 |
| pTH9073 |  | elt-1 end-3 | 75 | 1.67 | 2.2e-04 |
| Smad3\_3805 |  | daf-8 | 100 | 1.49 | 2.2e-04 |
| pTH6497 |  | lin-31 (0.67) | 119 | 1.39 | 2.3e-04 |
| LHX9\_2 |  | ceh-1 lin-39 ceh-14 ceh-31 | 115 | 1.41 | 2.4e-04 |
| Dlx2\_2273 |  | ceh-43 | 176 | 1.17 | 2.4e-04 |
| pTH5714 |  | nhr-239 | 97 | 1.50 | 2.4e-04 |
| TBX2\_f1 |  | ztf-6 tbx-39 | 106 | 1.45 | 2.4e-04 |
| GM12878\_PAX5N19\_HudsonAlpha |  | pax-3 pax-2 | 146 | 1.28 | 2.5e-04 |
| Hoxa10\_2318 |  | ceh-24 | 96 | 1.51 | 2.6e-04 |
| pTH9180 |  | let-381 (0.64) mel-28 mef-2 Y116A8C.22 Y61A9LA.9 | 141 | 1.29 | 2.7e-04 |
| Hoxa7\_3750 |  | lin-39 | 100 | 1.48 | 3.1e-04 |
| HES1\_f1 |  | lin-22 | 94 | 1.51 | 3.3e-04 |
| pTH9135 |  | pop-1 | 118 | 1.38 | 3.3e-04 |
| pTH6445 |  | ceh-5 | 66 | 1.74 | 3.3e-04 |
| pTH5250 |  | C48E7.11 | 113 | 1.41 | 3.4e-04 |
| pTH6106 |  | nhr-182 | 90 | 1.53 | 3.4e-04 |
| BARHL2\_1 |  | ceh-31 | 54 | 1.90 | 3.4e-04 |
| pTH10028 |  | nhr-204 | 90 | 1.53 | 3.6e-04 |
| Hoxb8\_3780 |  | lin-39 | 97 | 1.49 | 3.7e-04 |
| Mf28 |  | elt-1 | 93 | 1.51 | 3.9e-04 |
| Pou2f3\_3986 |  | ceh-18 | 96 | 1.49 | 4.0e-04 |
| V$GATA3\_01 |  | elt-1 | 80 | 1.60 | 4.2e-04 |
| MA0118.1 |  | ref-2 | 80 | 1.60 | 4.2e-04 |
| Plagl1\_0972 |  | Y53H1A.2 | 64 | 1.75 | 4.2e-04 |
| V$TAXCREB\_02 |  | zip-3 crh-1 | 91 | 1.52 | 4.2e-04 |
| pTH2283 |  | odd-2 | 69 | 1.69 | 4.3e-04 |
| V$BRN2\_01 |  | ceh-18 | 78 | 1.61 | 4.3e-04 |
| pTH9387 |  | C34D1.1 | 71 | 1.67 | 4.3e-04 |
| HSFY2\_1 |  | hsf-1 mab-3 | 105 | 1.44 | 4.4e-04 |
| MA0544.1 |  | lin-22 gei-11 | 83 | 1.57 | 4.5e-04 |
| Cutl1\_3494 |  | ceh-44 | 87 | 1.54 | 4.5e-04 |
| CrebA\_SANGER\_5\_FBgn0004396 |  | atf-6 fos-1 crh-1 C27D6.4 | 29 | 2.61 | 4.7e-04 |
| Hoxd11\_3873 |  | php-3 ceh-13 | 112 | 1.40 | 4.7e-04 |
| pTH5778 |  | egl-5 (0.57) | 103 | 1.45 | 4.8e-04 |
| pTH10015 |  | che-1 (0.52) ztf-28 ces-1 | 103 | 1.45 | 4.8e-04 |
| Hoxd10\_2368 |  | php-3 lin-39 | 67 | 1.71 | 5.0e-04 |
| pTH10042 |  | daf-12 nhr-5 | 102 | 1.45 | 5.2e-04 |
| pTH6281 |  | nhr-2 nhr-15 nhr-213 nhr-239 | 96 | 1.48 | 5.2e-04 |
| V$PAX5\_01 |  | pax-2 daf-8 F45H11.6 | 144 | 1.27 | 5.3e-04 |
| pTH5914 |  | attf-1 | 72 | 1.65 | 5.9e-04 |
| V$PAX2\_02 |  | pax-1 | 104 | 1.43 | 6.0e-04 |
| pTH9353 |  | ceh-51 | 95 | 1.48 | 6.3e-04 |
| RORG\_f1 |  | nhr-213 nhr-118 | 103 | 1.44 | 6.5e-04 |
| Nkx1-2\_3214 |  | ceh-30 | 159 | 1.21 | 6.8e-04 |
| pTH10772 |  | ceh-52 | 36 | 2.24 | 7.3e-04 |
| pTH9056 |  | bed-3 nhr-177 mbr-1 F26F4.8 | 97 | 1.46 | 7.4e-04 |
| POU2F2\_2 |  | unc-86 ceh-18 | 158 | 1.22 | 7.5e-04 |
| Hoxd13\_2356 |  | pal-1 | 121 | 1.35 | 7.6e-04 |
| pTH9222 |  | mel-28 | 93 | 1.48 | 7.8e-04 |
| Elf5 |  | C24A1.2 | 119 | 1.36 | 7.9e-04 |
| MA0163.1 |  | D1081.8 Y53H1A.2 C09F5.3 | 84 | 1.54 | 8.1e-04 |
| pTH1049 |  | elt-1 | 101 | 1.43 | 9.1e-04 |
| CUX1\_2 |  | ceh-48 | 71 | 1.63 | 9.1e-04 |
| V$GATA6\_01 |  | elt-1 | 105 | 1.41 | 9.2e-04 |
| pTH5781 |  | ceh-32 | 87 | 1.51 | 9.7e-04 |
| pTH5812 |  | ceh-14 | 68 | 1.66 | 9.8e-04 |
| pTH10031 |  | mbr-1 | 62 | 1.72 | 9.8e-04 |
| Hoxa11\_2218 |  | php-3 | 103 | 1.42 | 1.0e-03 |
| pTH10013 |  | nhr-168 | 58 | 1.77 | 1.0e-03 |
| Hmx3\_3490 |  | ceh-9 | 56 | 1.79 | 1.0e-03 |
| pTH3064 |  | crh-1 | 162 | 1.20 | 1.0e-03 |
| MA0095.2 |  | lsy-2 | 118 | 1.35 | 1.0e-03 |
| pTH3477 |  | daf-16 | 90 | 1.49 | 1.1e-03 |
| HeLa-S3\_ZNF274\_UCD |  | C28G1.4 | 100 | 1.43 | 1.1e-03 |
| disco-r-Cl1\_SANGER\_5\_FBgn0042650 |  | lin-31 (0.67) nhr-68 F55C5.11 | 100 | 1.43 | 1.1e-03 |
| Hoxa7\_2668 |  | lin-39 | 88 | 1.50 | 1.1e-03 |
| pTH8983 |  | tag-347 | 52 | 1.84 | 1.2e-03 |
| V$TBP\_01 |  | tbp-1 | 106 | 1.40 | 1.2e-03 |
| V$GATA1\_02 |  | elt-1 | 52 | 1.84 | 1.2e-03 |
| V$CREB\_Q4 |  | crh-1 | 98 | 1.44 | 1.2e-03 |
| pTH10024 |  | daf-19 F52B5.7 | 154 | 1.22 | 1.2e-03 |
| pTH9254 |  | mel-28 | 90 | 1.48 | 1.2e-03 |
| V$OCT1\_06 |  | ztf-9 ceh-18 | 87 | 1.50 | 1.2e-03 |
| pTH9164 |  | ceh-26 | 94 | 1.46 | 1.3e-03 |
| K562\_SP2\_HudsonAlpha |  | klf-2 | 87 | 1.50 | 1.3e-03 |
| ZBTB49\_1 |  | C46E10.9 aha-1 | 53 | 1.81 | 1.3e-03 |
| pTH10630 |  | lsy-27 | 34 | 2.24 | 1.3e-03 |
| pTH5924 |  | nhr-255 | 100 | 1.42 | 1.4e-03 |
| Bsx\_3483 |  | ceh-31 | 43 | 1.99 | 1.4e-03 |
| MA0027.1 |  | ceh-16 | 96 | 1.44 | 1.4e-03 |
| Hoxa6\_1040 |  | lin-39 | 99 | 1.43 | 1.4e-03 |
| pTH5882 |  | nhr-19 (0.8) nhr-69 (0.79) nhr-2 nhr-10 nhr-213 | 90 | 1.48 | 1.5e-03 |
| pTH2936 |  | nhr-239 | 89 | 1.48 | 1.5e-03 |
| pTH5922 |  | ceh-24 | 81 | 1.53 | 1.6e-03 |
| FOXG1\_2 |  | lin-31 (0.67) nhr-213 | 103 | 1.41 | 1.6e-03 |
| HXA7\_f1 |  | lin-39 elt-1 | 100 | 1.42 | 1.6e-03 |
| Pou3f4\_3773 |  | ceh-6 | 107 | 1.38 | 1.7e-03 |
| EN1\_4 |  | ceh-2 ceh-16 | 97 | 1.43 | 1.8e-03 |
| V$CEBP\_01 |  | C48E7.11 | 71 | 1.60 | 1.8e-03 |
| pTH9137 |  | nhr-65 | 139 | 1.26 | 1.8e-03 |
| Nkx6-3\_3446 |  | cog-1 (0.53) | 101 | 1.41 | 1.8e-03 |
| CG8765\_SANGER\_5\_FBgn0036900 |  | H20J04.3 | 99 | 1.42 | 1.9e-03 |
| Mv90 |  | mef-2 | 99 | 1.42 | 2.0e-03 |
| Pou3f1\_3819 |  | ceh-6 | 176 | 1.14 | 2.0e-03 |
| Dlx1\_1741 |  | ceh-43 | 174 | 1.15 | 2.1e-03 |
| MA0531.1 |  | F58G1.2 Y5F2A.4 | 82 | 1.51 | 2.2e-03 |
| MA0131.1 |  | F39B2.1 | 77 | 1.54 | 2.2e-03 |
| MA0470.1 |  | efl-1 F49E12.6 | 99 | 1.41 | 2.2e-03 |
| Hoxd1\_3448 |  | ceh-12 | 58 | 1.71 | 2.2e-03 |
| pTH9279 |  | Y116A8C.22 | 132 | 1.28 | 2.2e-03 |
| pTH8982 |  | ceh-48 | 105 | 1.38 | 2.2e-03 |
| Elf3\_3876 |  | C24A1.2 | 122 | 1.32 | 2.2e-03 |
| V$HOX13\_01 |  | lin-39 | 98 | 1.42 | 2.3e-03 |
| Nsy-7 |  | hmg-5 nsy-7 | 88 | 1.47 | 2.3e-03 |
| pTH6482 |  | ceh-19 | 88 | 1.47 | 2.3e-03 |
| Etv3 |  | lin-1 | 32 | 2.23 | 2.4e-03 |
| Hoxa4\_3426 |  | lin-39 | 95 | 1.43 | 2.5e-03 |
| Zic1\_0991 |  | T22C8.4 ref-2 | 63 | 1.65 | 2.5e-03 |
| pTH9189 |  | dmd-3 ceh-18 | 117 | 1.33 | 2.5e-03 |
| Hoxb3\_1720 |  | lin-39 | 92 | 1.44 | 2.7e-03 |
| V$OCT1\_03 |  | ceh-18 | 61 | 1.66 | 2.8e-03 |
| pTH6569 |  | ceh-43 | 40 | 1.98 | 2.8e-03 |
| pTH6641 |  | lin-31 (0.67) | 83 | 1.49 | 2.9e-03 |
| Gata5\_3768 |  | elt-1 | 102 | 1.39 | 2.9e-03 |
| Six4\_2860 |  | ceh-32 | 135 | 1.26 | 2.9e-03 |
| pTH6449 |  | ceh-43 | 58 | 1.69 | 3.0e-03 |
| pTH3046 |  | Y116A8C.22 | 81 | 1.50 | 3.1e-03 |
| Poxm\_SOLEXA\_5\_FBgn0003129 |  | pax-2 | 83 | 1.48 | 3.3e-03 |
| pTH6636 |  | egl-5 (0.57) | 98 | 1.40 | 3.3e-03 |
| Nkx6-1\_2825 |  | cog-1 (0.53) | 101 | 1.39 | 3.3e-03 |
| pTH5267 |  | hlh-32 hlh-16 ngn-1 | 79 | 1.51 | 3.4e-03 |
| rn\_SOLEXA\_5\_FBgn0259172 |  | lin-29 | 57 | 1.69 | 3.7e-03 |
| Hoxb4\_2627 |  | lin-39 | 96 | 1.41 | 3.8e-03 |
| K562\_ZBTB7A\_HudsonAlpha |  | ZC328.2 | 78 | 1.51 | 3.8e-03 |
| pTH4325 |  | ceh-18 | 121 | 1.30 | 3.8e-03 |
| Elf4 |  | C24A1.2 | 64 | 1.61 | 4.0e-03 |
| pTH9381 |  | ceh-18 | 180 | 1.12 | 4.0e-03 |
| pTH1014 |  | atf-5 (0.63) | 33 | 2.13 | 4.0e-03 |
| V$DELTAEF1\_01 |  | ztf-6 hlh-2 | 89 | 1.44 | 4.0e-03 |
| Hoxc4\_3491 |  | lin-39 | 91 | 1.43 | 4.1e-03 |
| pTH9043 |  | sem-2 | 114 | 1.33 | 4.3e-03 |
| pTH9326 |  | nhr-122 | 104 | 1.37 | 4.3e-03 |
| MA0032.1 |  | let-381 (0.64) | 60 | 1.64 | 4.5e-03 |
| PROX1\_1 |  | crh-1 ceh-26 | 80 | 1.48 | 4.6e-03 |
| Evx1\_3952 |  | ceh-53 (-0.56) | 158 | 1.18 | 4.7e-03 |
| pTH5887 |  | lin-39 | 82 | 1.47 | 4.7e-03 |
| HLH10 |  | unc-120 (0.79) hlh-10 | 138 | 1.24 | 4.8e-03 |
| pTH6436 |  | ceh-53 (-0.56) | 56 | 1.68 | 4.9e-03 |
| PAX5\_si |  | D1081.8 pax-2 F45H11.6 | 96 | 1.40 | 4.9e-03 |
| pTH3819 |  | ceh-18 | 97 | 1.39 | 5.2e-03 |
| V$S8\_01 |  | ceh-45 | 180 | 1.11 | 5.3e-03 |
| pTH9177 |  | hsf-1 F10B5.3 Y53C10A.3 | 121 | 1.29 | 5.3e-03 |
| Vax2\_3500 |  | C02F12.10 | 89 | 1.43 | 5.4e-03 |
| Pbx1\_3203 |  | ceh-20 | 107 | 1.35 | 5.4e-03 |
| FLI1\_f1 |  | lin-1 | 88 | 1.43 | 5.4e-03 |
| Pknox2\_3077 |  | ceh-32 | 152 | 1.20 | 5.4e-03 |
| pTH6562 |  | ceh-5 | 57 | 1.66 | 5.4e-03 |
| pTH2846 |  | lin-31 (0.67) | 128 | 1.27 | 5.7e-03 |
| pTH8649 |  | mbr-1 | 65 | 1.58 | 5.7e-03 |
| pTH8996 |  | hlh-8 (0.76) sma-4 | 96 | 1.39 | 5.7e-03 |
| V$YY1\_01 |  | lsy-2 | 112 | 1.32 | 5.8e-03 |
| pTH9335 |  | mel-28 | 82 | 1.46 | 5.9e-03 |
| V$TCF11\_01 |  | skn-1 | 164 | 1.16 | 6.1e-03 |
| V$IK2\_01 |  | F26F4.8 | 93 | 1.40 | 6.7e-03 |
| Pou2f1\_3081 |  | ceh-18 | 58 | 1.63 | 6.8e-03 |
| pTH10654 |  | ceh-90 lsy-27 | 78 | 1.47 | 7.0e-03 |
| pTH9934 |  | Y53H1A.2 | 138 | 1.23 | 7.2e-03 |
| MA0161.1 |  | nfi-1 | 58 | 1.63 | 7.4e-03 |
| FLI1\_4 |  | lin-1 | 86 | 1.43 | 7.6e-03 |
| Atf1\_3026 |  | crh-1 | 97 | 1.37 | 7.8e-03 |
| Meox1\_2310 |  | ceh-31 | 166 | 1.15 | 8.3e-03 |
| pTH9082 |  | mab-23 | 125 | 1.27 | 8.3e-03 |
| I$DFD\_01 |  | lin-39 | 86 | 1.42 | 8.5e-03 |
| pTH9260 |  | mel-28 | 104 | 1.34 | 8.6e-03 |
| Irx5\_2385 |  | irx-1 | 87 | 1.41 | 9.6e-03 |
| V$EN1\_01 |  | ceh-16 | 86 | 1.41 | 1.1e-02 |
| tgo\_ss\_SANGER\_5\_FBgn0015014 |  | aha-1 | 84 | 1.42 | 1.1e-02 |
| pTH10805 |  | ztf-16 | 88 | 1.39 | 1.2e-02 |
| pTH6268 |  | ceh-2 | 65 | 1.53 | 1.2e-02 |
| pTH10650 |  | nhr-153 | 168 | 1.14 | 1.3e-02 |
| pTH9880 |  | end-1 | 59 | 1.57 | 1.3e-02 |
| pTH9884 |  | tbx-39 | 90 | 1.38 | 1.4e-02 |
| Hoxa3\_2783 |  | lin-39 | 86 | 1.40 | 1.4e-02 |
| pTH9913 |  | skn-1 fos-1 | 104 | 1.32 | 1.4e-02 |
| CENPB\_1 |  | F21D5.4 | 87 | 1.39 | 1.4e-02 |
| Barx2\_3447 |  | ceh-43 | 101 | 1.33 | 1.5e-02 |
| Barx1\_2877 |  | ceh-43 | 158 | 1.16 | 1.6e-02 |
| Hoxa2\_3079 |  | lin-39 | 83 | 1.40 | 1.7e-02 |
| pTH8985 |  | athp-1 | 154 | 1.17 | 1.7e-02 |
| Hmbox1\_2674 |  | hmbx-1 | 74 | 1.45 | 1.8e-02 |
| Osr2\_1727 |  | odd-1 odd-2 | 69 | 1.47 | 2.0e-02 |
| pTH8556 |  | pax-2 | 73 | 1.45 | 2.0e-02 |
| pTH9220 |  | mbr-1 | 98 | 1.33 | 2.0e-02 |
| V$NCX\_01 |  | ceh-19 | 157 | 1.16 | 2.0e-02 |
| Hlxb9\_3422 |  | ceh-12 | 159 | 1.15 | 2.1e-02 |
| V$CETS1P54\_02 |  | C52B9.2 | 109 | 1.29 | 2.1e-02 |
| Irx3\_0920 |  | irx-1 | 81 | 1.40 | 2.2e-02 |
| I$UBX\_01 |  | lin-39 | 146 | 1.18 | 2.2e-02 |
| pTH10769 |  | Y48G1C.6 | 98 | 1.32 | 2.5e-02 |
| Emx2\_3420 |  | ceh-2 | 153 | 1.16 | 2.8e-02 |
| pTH9256 |  | ceh-18 | 183 | 1.08 | 3.2e-02 |
| YMR043W\_831 |  | unc-120 (0.79) | 97 | 1.31 | 3.2e-02 |
| pTH9219 |  | xbp-1 C01B12.2 | 61 | 1.49 | 3.3e-02 |
| Hoxb5\_3122 |  | lin-39 | 83 | 1.37 | 3.3e-02 |
| pTH5916 |  | efl-2 | 73 | 1.41 | 3.6e-02 |
| pTH3997 |  | C04F5.9 | 135 | 1.20 | 3.8e-02 |
| Gsh2\_3990 |  | ceh-31 | 83 | 1.36 | 3.9e-02 |
| pTH10041 |  | ztf-29 | 50 | 1.57 | 4.0e-02 |
| pTH10768 |  | med-2 | 138 | 1.18 | 4.8e-02 |

### Correlated (and anti-correlated) transcription factors

|  |  |
| --- | --- |
| **Transcription factor** | **Correlation** |
| unc-98 | 0.91 |
| hlh-1 | 0.87 |
| rnt-1 | 0.84 |
| K05F1.5 | 0.82 |
| pat-9 | 0.80 |
| nhr-19 | 0.80 |
| unc-120 | 0.79 |
| K02D7.2 | 0.79 |
| nhr-69 | 0.79 |
| hlh-8 | 0.76 |
| ceh-49 | 0.73 |
| mls-1 | 0.73 |
| nhr-57 | 0.72 |
| dsc-1 | 0.72 |
| camt-1 | 0.71 |
| nhr-87 | 0.69 |
| lin-31 | 0.67 |
| ceh-33 | 0.65 |
| hlh-4 | 0.65 |
| hnd-1 | 0.64 |
| ehn-3 | 0.64 |
| let-381 | 0.64 |
| atf-5 | 0.63 |
| mec-3 | 0.62 |
| moe-3 | 0.62 |
| tbx-32 | -0.42 |
| madf-7 | -0.43 |
| Y57A10A.31 | -0.43 |
| xnd-1 | -0.45 |
| wrm-1 | -0.45 |
| odd-1 | -0.47 |
| zim-3 | -0.47 |
| F49E8.2 | -0.47 |
| nhr-155 | -0.48 |
| gla-3 | -0.50 |
| elt-2 | -0.51 |
| pqn-75 | -0.51 |
| hmg-3 | -0.52 |
| ztf-4 | -0.52 |
| D2030.7 | -0.54 |
| K11H3.4 | -0.55 |
| ceh-53 | -0.56 |
| ccch-3 | -0.56 |
| nhr-80 | -0.56 |
| elt-7 | -0.57 |
| cep-1 | -0.58 |
| ztf-15 | -0.59 |
| snu-23 | -0.59 |
| nhr-246 | -0.62 |
| pzf-1 | -0.64 |

### ChIP peaks enriched

|  |  |  |  |  |
| --- | --- | --- | --- | --- |
| **Gene** | **Experiment** | **Number of upstream peaks** | **Enrichment** | **FDR corrected p** |
| egl-5 | EGL-5\_Larvae-L3-stage | 79 | 2.52 | 4.4e-14 |
| nhr-129 | NHR-129\_Larvae-L2-stage | 62 | 2.33 | 5.6e-09 |
| C34F6.9 | C34F6.9\_Larvae-L2-stage | 42 | 2.41 | 4.6e-06 |
| lsy-2 | LSY-2\_Larvae-L1-stage | 46 | 2.09 | 5.5e-05 |
| sem-4 | SEM-4\_Larvae-L2-stage | 40 | 2.09 | 2.9e-04 |
| lsy-2 | LSY-2\_Fed-L1-stage-larvae | 56 | 1.75 | 5.4e-04 |
| fkh-2 | FKH-2\_Larvae-L3-stage | 17 | 2.90 | 3.9e-03 |
| nhr-6 | NHR-6\_Larvae-L2-stage | 30 | 1.98 | 9.3e-03 |
| nfya-1 | NFYA-1\_Late-Embryos | 29 | 1.82 | 4.0e-02 |
